# Phase variation and microevolution at homopolymeric tracts in *Bordetella pertussis*

**DOI:** 10.1186/1471-2164-8-122

**Published:** 2007-05-17

**Authors:** Emily B Gogol, Craig A Cummings, Ryan C Burns, David A Relman

**Affiliations:** 1 Department of Microbiology and Immunology, Stanford University School of Medicine, Stanford, California 94305, USA; 2 Department of Medicine, Stanford University School of Medicine, Stanford, California 94305, USA; 3 VA Palo Alto Health Care System, Palo Alto, California 94304, USA; 4 2030 3rd. St., Unit #2, San Francisco, CA 94107, USA; 5 Present address : University of California San Francisco, Biomedical Sciences Graduate Program, 513 Parnassus Ave., Room HSE-1285, San Francisco, CA, 94143, USA

## Abstract

**Background:**

*Bordetella pertussis*, the causative agent of whooping cough, is a highly clonal pathogen of the respiratory tract. Its lack of genetic diversity, relative to many bacterial pathogens, could limit its ability to adapt to a hostile and changing host environment. This limitation might be overcome by phase variation, as observed for other mucosal pathogens. One of the most common mechanisms of phase variation is reversible expansion or contraction of homopolymeric tracts (HPTs).

**Results:**

The genomes of *B. pertussis *and the two closely related species, *B. bronchiseptica *and *B. parapertussis*, were screened for homopolymeric tracts longer than expected on the basis of chance, given their nucleotide compositions. Sixty-nine such HPTs were found in total among the three genomes, 74% of which were polymorphic among the three species. Nine HPTs were genotyped in a collection of 90 geographically and temporally diverse *B. pertussis *strains using the polymerase chain reaction/ligase detection reaction (PCR/LDR) assay. Six HPTs were polymorphic in this collection of *B. pertussis *strains. Of note, one of these polymorphic HPTs was found in the *fimX *promoter, where a single base insertion variant was present in seven strains, all of which were isolated prior to introduction of the pertussis vaccine. Transcript abundance of *fimX *was found to be 3.8-fold lower in strains carrying the longer allele. HPTs in three other genes, *tcfA*, *bapC*, and BP3651, varied widely in composition across the strain collection and displayed allelic polymorphism within single cultures.

**Conclusion:**

Allelic polymorphism at homopolymeric tracts is common within the *B. pertussis *genome. Phase variability may be an important mechanism in *B. pertussis *for evasion of the immune system and adaptation to different niches in the human host. High sensitivity and specificity make the PCR/LDR assay a powerful tool for investigating allelic variation at HPTs. Using this method, allelic diversity and phase variation were demonstrated at several *B. pertussis *loci.

## Background

*Bordetella pertussis *causes whooping cough, a highly communicable disease that killed roughly 279,000 people and infected 17.6 million people globally in a recent typical year [1]. *B. pertussis *and the closely related human- and sheep-adapted species, *B. parapertussis*, have diverged independently by genome decay from a putative common ancestor that they share with *B. bronchiseptica*, which has a broader host range, and unlike the other two species, causes chronic infections [2-4]. Re-emergence of pertussis in certain vaccinated populations [5,6] suggests that *B. pertussis *may be adapting to vaccine-induced host immunity [5]. In support of this hypothesis, shifts in the allelic frequencies of genes encoding at least three vaccine components (pertussis toxin, pertactin, and fimbriae) have been documented in *B. pertussis *from Finland, Sweden, France and the Netherlands [5,7-9]. However, multilocus enzyme electrophoresis (MLEE) [10], comparative genome hybridization (CGH) [11] and multilocus sequence typing (MLST) of seven housekeeping genes [4] have established that *B. pertussis *is highly clonal with nearly invariant genome content. The apparent scarcity of variation in the *B. pertussis *genome is unusual among bacterial pathogens, in which extensive genomic plasticity is thought to contribute to host immune evasion [12].

Phase variation is one means by which *B. pertussis *might adapt to host immune surveillance and vaccine-induced immune responses without loss or acquisition of genomic fragments [13]. In a number of pathogenic bacteria, phase variation influences expression of virulence phenotypes and creates phenotypic diversity in clonal populations (reviewed in [14,15]). Phase variation can also influence the affinity of pathogens for different host anatomical niches. For example, variable expression of colony opacity (Opa) proteins changes the tropism of *Neisseria *for human epithelium, endothelium, and phagocytic cells [16].

The genetic mechanism of phase variation in many cases is expansion and contraction of nucleotide repeat sequences (reviewed in [17,18]). Homopolymeric nucleotide tracts (HPTs) frequently grow or shrink, with longer HPTs more prone to slippage. By searching bacterial genome sequences for unusually long HPTs, putative phase-variable genes have been identified, and in some cases, validated [19-22].

*B. pertussis *undergoes variation between virulent and avirulent phases [23]. The molecular mechanism of this phenotypic switch is expansion and contraction of an HPT in *bvgS *[24], which encodes a key regulator of virulence gene expression (reviewed in [25]). In addition, phase-variable expression of two fimbrial major subunit genes, *fim2 *and *fim3*, has been attributed to variations in HPTs located within the promoter regions of these genes that lead to altered transcript abundance [26,27]. In this study, we sought to identify additional *B. pertussis *candidate phase-variable genes, particularly those with potential roles in pathogenesis. Furthermore, we sought to test whether these genes were polymorphic in the *B. pertussis *population and, specifically, whether they were phase-variable over the short time period that characterizes an infectious cycle.

In order to measure HPT tract length in a rapid, sensitive, and accurate manner in a large collection of *B. pertussis *strains, the polymerase chain reaction/ligase detection reaction (PCR/LDR) was employed. This assay has been used to study genomic repeat instability in human tumors [28] and has been applied to the identification of rifampin-resistant *Mycobacterium tuberculosis *strains [29]. The ability of PCR/LDR to identify rare genotypic variants in a population, and its high fidelity, make this method well suited for detection of HPT-associated phase variation events. Using this method, HPT length polymorphisms were demonstrated at six out of nine putative phase-variable loci, among a collection of 90 *B. pertussis *isolates. Two of these loci, both encoding Bvg-activated surface proteins, were found to exhibit rapid and reversible mutations. Phase variation in *B. pertussis *is more common than previously recognized and may be a significant source of phenotypic diversity in this pathogen.

## Results

### Validation of PCR/LDR for detection of HPT alleles in bacterial genomes

Variation between virulent and avirulent phenotypic phases in *B. pertussis *has been attributed to a polymorphism within an HPT located in the coding region of the virulence regulatory sensor kinase gene, *bvgS *[24]. Virulent strains (e.g., BP370) have 6 cytosines (C_6_) in this HPT and produce full-length BvgS. The HPT of avirulent derivative strains (e.g., BP369) has 7 cytosines (C_7_), leading to a translational frameshift that truncates BvgS, rendering it non-functional [24].

**Figure 1:**
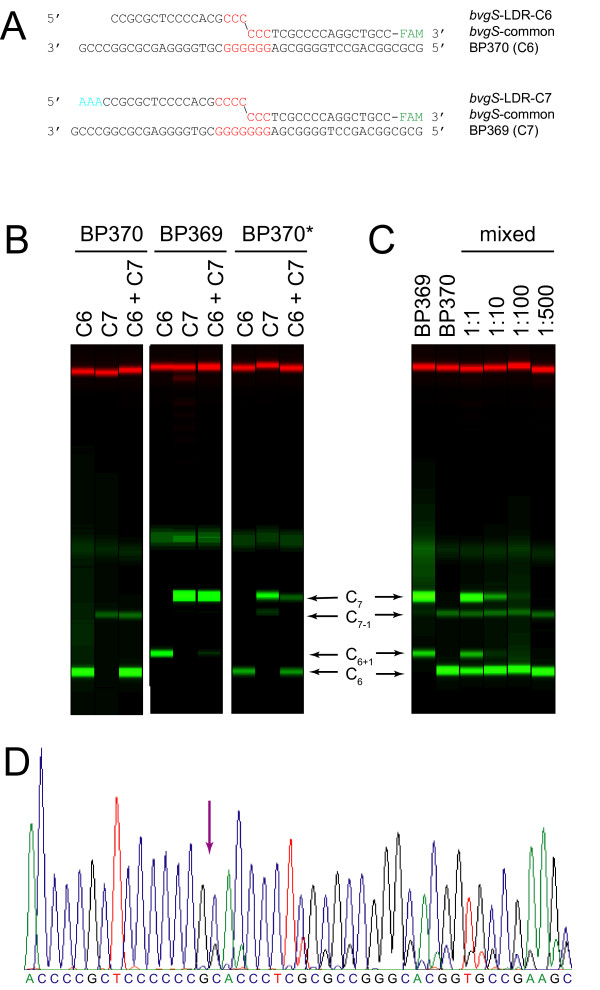
**Detection by PCR/LDR of HPT alleles in *bvgS***. (**A**) Schematic of BP370 and BP369 genomic DNA templates aligned with LDR oligonucleotides. Purple text denotes the HPT; green star indicates the FAM fluorophore; cyan text shows non-hybridizing bases used for size discrimination of oligos. HPT length is six cytosines (C_6_) in BP370, and seven cytosines (C_7_) in BP369. Covalent bonds (diagonal lines) are catalyzed by DNA ligase only if the discriminating and common oligonucleotides are immediately adjacent when hybridized to the template. (**B**) Raw capillary electrophoresis data for ligation products (green) and molecular weight standards (red) displayed as if an electrophoretic gel image. Source of template genomic DNA is indicated across the top of the panel. BP370* is a culture derived from BP370 that contains a minority population of C_7 _phase variants. All reactions contained common oligonucleotide; discriminating oligonucleotides are indicated above each lane (C_6 _and C_7 _indicate uniplex reactions, C_6 _+ C_7 _indicates multiplex). Ligation products (arrows) are labeled as follows. C_7 _and C_6 _indicate the products formed by ligation of the common oligonucleotide to the C_7 _and C_6 _discriminating oligonucleotides, respectively. C_6+1 _denotes the product formed by ligation of the common oligonucleotide to a synthesis artifact in the C_6 _discriminating oligonucleotide preparation that has one extra 3' C. Similarly, C_7-1 _denotes the product formed by ligation of the common oligonucleotide to a synthesis artifact in the C_7 _discriminating oligonucleotide preparation that has one fewer 3' C. This notation is used throughout the figures of this manuscript. (**C**) Raw capillary electrophoresis displayed as in panel B. All lanes are multiplex reactions using C_6 _and C_7 _discriminating oligos with the genomic DNA template indicated above each lane. Lanes 3–6 show the ratio of PCR amplified *bvgS *from BP369 to *bvgS *from BP370. Ligation products are labeled as in panel B. (**D**) Partial trace of directly sequenced *bvgS *PCR product from BP370*. Purple arrow indicates the point in the sequence trace after which peak shadowing, indicative of a mixture of HPT alleles, can be observed.

In order to test the utility of PCR/LDR for detecting HPT polymorphisms, oligonucleotides were designed for discrimination of the C_6 _and C_7 _alleles of *bvgS *(Figure 1A). A *bvgS *fragment containing the HPT was amplified by PCR using a high-fidelity, thermostable DNA polymerase (*Pfu *Ultra). Each LDR reaction contained the amplified template DNA, thermostable *Taq *DNA ligase, a 5-carboxyfluorescein (FAM)-labeled "common" oligonucleotide, and one or more unlabeled "discriminating" oligonucleotides that differ in length from each other by at least three nucleotides so as to enhance electrophoretic separation of the ligation products. The common oligonucleotide was designed to be complementary to 3 bases of the HPT plus 15 bases of downstream sequence. Both the C_6 _and C_7 _discriminating oligonucleotides were complementary to 14 bases of the upstream flanking sequence, but they differed in the number of repeated bases at the 3' end: the C_6 _discriminating oligonucleotide had three repeated bases, and the C_7 _discriminating oligonucleotide had four. When the sum of the repeated bases at the 3' end of the discriminating oligonucleotide and the 5' end of the common oligonucleotide equals the number of repeated bases in the template HPT, the alignment of the oligos on the template favors the formation of a single-stranded ligation product with diagnostic size (Figure 1A).

As expected, PCR/LDR analysis of the virulent BP370 strain with the C_6 _discriminating oligonucleotide yielded an abundant ligation product (Figure 1B). However, PCR/LDR with the C_7 _discriminating oligonucleotide preparation also produced a ligation product, albeit more faint. This finding can be attributed to oligonucleotide synthesis errors (see capillary electrophoresis data in Additional file 1) that cause a fraction of the oligonucleotides (denoted "C_7-1_") in the HPLC-purified synthesis product to contain one fewer cytosine at the 3' end. These rare synthesis artifacts can, in the presence of a C_6 _template, form a detectable ligation product. This interpretation is further supported by the lower mass of the C_7-1 _ligation product compared to the C_7 _ligation product that would be formed by the full-length C_7 _oligonucleotide in the presence of a C_7 _template. Conversely, PCR/LDR of the avirulent parent, BP369, generated an abundant ligation product with the C_7 _discriminating oligonucleotide, as expected. However, as above, a faint ligation product produced with the C_6 _discriminating oligonucleotide preparation, with higher mass than expected for the C_6 _product, can be attributed to synthesis errors that result in rare oligonucleotides (denoted "C_6+1_") with one additional cytosine at the 3' end. Ligation products formed by synthesis artifacts could be easily distinguished from legitimate products by their characteristic electrophoretic mobility. Equivalent ligation products were obtained whether reactions contained one (uniplex) or both (multiplex) discriminating oligonucleotides (Figure 1B).

In order to examine the ability of PCR/LDR to detect infrequent HPT alleles in a mixed population of templates, *bvgS *PCR product from BP369 was mixed with *bvgS *PCR product from BP370 in a series of dilutions (Figure 1C). The C_7 _allele from BP369 could be detected in a multiplex reaction when present at 100-fold lower concentration than the C_6 _allele, but not at 500-fold lower concentration. This sensitivity is similar to that observed for PCR/LDR detection of single nucleotide HPT variants on synthetic templates [28]. Thus, this method can detect phase variants that comprise as little as 1% of the sample template.

To assess the sensitivity of PCR/LDR in identifying a mixture of alleles in a bacterial sample, genomic DNA was prepared from a culture of BP370*, a strain that was isolated during a single passage of BP370 on BG blood agar. As the result of spontaneous mutation, BP370* contains a mixture of virulent and avirulent phase variants, as determined by plating on BG blood agar. Multiplex PCR/LDR of this sample yielded ligation products with both the C_6 _and C_7 _discriminating oligonucleotides, verifying that the genomic DNA preparation from this strain contained a mixture of C_6 _and C_7 _*bvgS *alleles (Figure 1B). This result was further validated by DNA sequencing of the *bvgS *PCR product from the same DNA preparation (Figure 1D). Thus, cultures composed of mixed populations with distinct HPT alleles, such as those that arise during laboratory passage of *B. pertussis*, are readily identifiable by PCR/LDR.

The rate of repeat expansion and contraction due to DNA polymerase slippage rises with increasing repeat number [30]. Therefore, high-fidelity PCR amplification is critical to this allelic detection scheme, especially for long HPT tracts. In order to asses the fidelity of PCR/LDR, a region of *bapC *containing a G_10 _tract was amplified using either high-fidelity *Pfu *DNA polymerase or the relatively lower fidelity *Taq *DNA polymerase, then screened by LDR (as described below). After 30 cycles of PCR, LDR of the *Pfu*-amplified template showed no tract length variation, but LDR of the *Taq*-amplified template detected tracts of 9, 10, and 11 guanosines (Additional file 2). Subsequent rounds of PCR product dilution and re-amplification with *Pfu *polymerase did not yield any detectable HPT variants (contractions) until the third round (90 cycles of PCR); tract expansions were not observed even after 120 cycles. Therefore, in a standard PCR/LDR experiment (30 cycles of PCR), there is negligible variation in HPTs 10 bases or shorter due to polymerase replication error when *Pfu *DNA polymerase is utilized. These results indicate that tract length heterogeneity detected by PCR/LDR as performed in this study accurately reflects HPT length heterogeneity *in vivo*.

### Identification of putative phase-variable genes in *Bordetella *genomes

**Figure 2:**
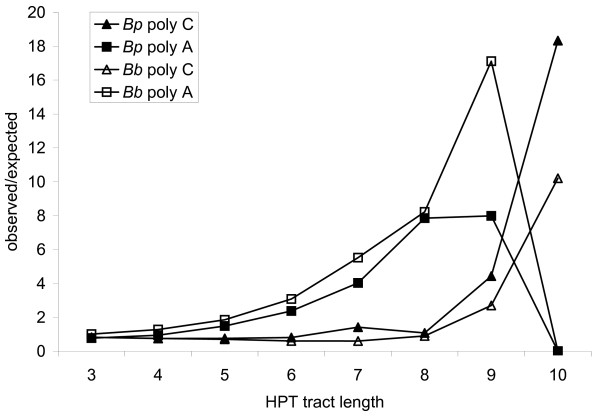
**Observed/expected plots of different length HPTs in the *B. pertussis *and *B. bronchiseptica *genomes**. Expected frequencies of A and C HPTs in the *B. pertussis *Tohama I (*Bp*) and *B. bronchiseptica *RB50 (*Bb*) genomes were calculated for a range of tract lengths using a second order Markov model. For each tract length, observed/expected values were determined by dividing the actual number of HPTs greater than or equal to that tract length by the expected number. The observed/expected value for A HPTs drops to zero at tract length of 10 because neither genome harbors an A HPT with more than 9 bases.

To identify putative phase-variable genes in *B. pertussis*, the genome sequence of the Tohama I strain [3] was screened for HPTs. Statistical analysis of sequence composition with a second order Markov model indicated that C/G HPTs more than 8 bases long, and A/T HPTs more than 5 bases long were observed more frequently than would be expected by chance in the *B. pertussis *and *B. bronchiseptica *genome sequences (Figure 2). To be conservative, the threshold for calling A/T HPTs was raised to greater than 7, due to the relative stability of shorter repeats in experimental systems [31]. Although shorter HPTs are known to be phase-variable in *B. pertussis *(e.g., C_6 _in *bvgS*), only genes associated with HPTs exceeding these conservative thresholds were classified as putative phase-variable genes in this computational screen. Because only a single *B. pertussis *genome sequence is available, it was not possible to search for polymorphic HPTs between strains of this species. However, because *B. pertussis *and *B. parapertussis *recently evolved from *B. bronchiseptica *[2-4], orthologous sequences of these three species are highly conserved. Of particular relevance to this study, single nucleotide indels between the *B. pertussis *and *B. bronchiseptica *genomes are found at fewer than 1 in 10,000 bases. Therefore, any HPTs that are polymorphic among these three species were strong candidates for phase-variable loci. In accordance with this logic, the *B. parapertussis *and *B. bronchiseptica *genomes [3] were also screened for HPTs, and for each tract, the orthologous locus was examined in all three species. This screen resulted in the identification of 69 HPT loci among the three species (Table 1; Additional files 3, 4, and 5). Because sequence composition differs in mobile genetic elements, HPTs in prophage or insertion elements were excluded from further analysis.

**Table 1:** Significant HPTs in *B. pertussis*, *B. bronchiseptica*, and *B. Parapertussis*

	***B. pertussis *Tohama I**		***B. bronchiseptica *RB50**		***B. parapertussis *12822**							
**HPT**^***a***^	**ORF**^***b***^	**Allele**^***c***^	**ORF**	**Allele**	**ORF**	**Allele**	**Gene**	**Base**	**Poly-morphic**^***d***^	**Loc**^***e***^	**Promoter overlap**^***f***^	**Functional category**^***g***^

11	BP2991	7	BB4384	9	BPP3911	9		A/T	y	I	L	Biosynthesis of cofactors, carriers
12	BP3682	8	BB0097	8	BPP0098	8		A/T	n	I	M	Hypothetical protein
13			BB0618, BB0619	8	BPP0612, BPP0613	7		A/T	y	I	H	Global regulatory functions
14			BB0622	8	BPP0616	8		A/T	n	I	M	Conserved hypothetical
15	BP3151	9	BB0875	8	BPP0790	8		A/T	y	I	L	Cell envelope
16			BB0999	8	BPP0906	8		A/T	n	I	L	Transport/binding proteins
**17**	BP0880	7	BB1564	8	BPP2167	7		A/T	y	I	L	Periplasmic/exported/lipoproteins
18	BP2792	8	BB1918	8	BPP2471	8		A/T	n	I	H	Hypothetical protein
19	BP2908	8	BB1935	8	BPP2488	8	*aroG*	A/T	n	I	L	Amino acid biosynthesis
20	BP2735	8	BB2036	8	BPP2594	8		A/T	n	I	H	Conserved hypothetical
21	BP2539	8	BB2083	8	BPP2640	8		A/T	n	I	L	Cell envelope
23	BP1487	8	BB2136	8	BPP1948	8	*smoM*^*h*^	A/T	n	I	M	Transport/binding proteins
24	BP1419	8	BB2605	8	BPP1527	8	*rpsB*	A/T	n	I	M	Ribosome constituents
25	BP1245	9	BB3248	8	BPP1860	11		A/T	y	I	M	Conserved hypothetical
26	BP1231	8	BB3262	8	BPP1846	8		A/T	n	I	H	Cell envelope
27	BP2399	8	BB3715	8	BPP3264	7		A/T	y	I	L	Global regulatory functions
28	BP2425	8	BB3740	8	BPP3289	9		A/T	y	I	M	Periplasmic/exported/lipoproteins
29	BP0858	9	BB3824	8	BPP3373	9		A/T	y	I	H	Hypothetical protein
30	BP0856	8	BB3826	8	BPP3376	10	*bfrD*	A/T	y	I	L	Adaptation
31	BP3399	8	BB3987	8	BPP3552	8		A/T	n	I	M	Conserved hypothetical
32			BB4499	8	BPP4026	8		A/T	y	I	H	Conserved hypothetical
**33**	BP0347	8	BB4655	8	BPP4185	8	*bhuR*	A/T	n	I	K	Adaptation
34	BP3870	8	BB5012	8	BPP4424	8		A/T	n	I	M	Hypothetical protein
41	BP2506, BP2507	9	BB3941, BB3942	9	BPP3494	9		A/T	n	I	N	Macromolecule synthesis, modification
43	BP2863	9	BB1178	9	BPP0966	9		A/T	n	I	H	Conserved hypothetical
50	BP0939	8						A/T	y	I	L	Conserved hypothetical
51	BP0964	8	BB3455	9	BPP3115	9		A/T	y	I	L	Hypothetical protein
52	BP0966	8	BB3453	9	BPP1655	9	*sbp*	A/T	y	I	M	Transport/binding proteins
56	BP2936	8	BB1280	8	BPP1064	9		A/T	y	I	M	Periplasmic/exported/lipoproteins
60	BP0598	6	BB0289	6	BPP0286	8		A/T	y	I	L	Conserved hypothetical
64			BB1238	7	BPP1024	8		A/T	y	I	M	Energy metabolism, carbon
68	BP2084	7	BB3340	7	BPP1768	8		A/T	y	I	H	Conserved hypothetical
69	BP1811	6	BB3216	6	BPP1892	8	*kdgT*	A/T	y	I	M	Transport/binding proteins
70	BP1781	6	BB2260	7	BPP2012	8		A/T	y	C		Conserved hypothetical
73					BPP2251	8	*bapA*	A/T	y	I	L	Cell envelope
76	BP1893, BP1894^i^	A_2_CA_6_	BB2980, BB2979^i^	A_2_CA_6_	BPP3014, BPP3013^i^	9		A/T	y	I	H	Cons. hypo.; Global reg. functions
79	BP3852	nr^j^	BB4994	7	BPP4406	8	*katA*	A/T	y	I	L	Protection responses
83			BB3111, BB3112^i^	8	BPP1616, BPP1617^i^	8		A/T	n	I	L	Cell envelope; hypothetical protein
84	tRNA-Asn, tRNA-Thr^i^	8	tRNA-Asn, tRNA-Thr^i^	8	tRNA-Asn, tRNA-Thr^i^	8		A/T	n	I	U	Macromolecule synthesis, modification
1			BB0357	11	BPP0354	8		C/G	y	I	M	Periplasmic/exported/lipoproteins
2			BB1186	10	BPP0974	7		C/G	y	I	M	Periplasmic/exported/lipoproteins
3	BP1568	13	BB1658	22	BPP2262	C_6_TC_3_	*fim3*	C/G	y	I	K	Cell envelope
**4**	BP2738	11	BB2033	7	BPP2591	7	*bapC*^*h*^	C/G	y	C		Cell envelope
5			BB3110	17	BPP1618	nr		C/G	y	C		Cell envelope
**6**	BP1201	9	BB3291	17			*tcfA*	C/G	y	C		Cell envelope
7			BB3425	15	BPP1683	10	*fimN*	C/G	y	I	K	Cell envelope
**8**	BP2674	7	BB3426	13	BPP1682	9	*fimX*	C/G	y	I	K	Cell envelope
9	BP1119	12	BB3674	20	BPP3222	C_9_TC_8_	*fim2*	C/G	y	I	K	Cell envelope
10	BP3037, BP3038	11	BB4210, BB4211	11	BPP3764, BPP3765	9		C/G	y	I	N	Hypothetical protein
22	BP1487	9	BB2136	C_5_GC_3_	BPP1948	C_5_GC_3_	*smoM*^*h*^	C/G	y	C		Transport/binding proteins
35	BP0146	15	BB4482	6	BPP4009	6		C/G	y	I	H	Hypothetical protein
36	BP1766	10	BB2245	G_6_TG_3_	BPP1997	8		C/G	y	I	N	Central intermediary metabolism
37	BP1879	10	BB2993	3	BPP3027	2	*fhaB*	C/G	y	C		Cell envelope
39	BP1961	11	BB1784	nr	BPP2333	nr		C/G	y	I	H	Energy metabolism, carbon
40	BP2483	10	BB3920	C_2_AC_5_	BPP3471	C_2_AC_5_	*kdpD*	C/G	y	C		Global regulatory functions
42	BP2595	12	BB2360	G_3_TG_3_	BPP1295	G_3_TG_3_		C/G	y	C		Macromolecule degradation
44	BP2896	9	BB1947	8	BPP2500	7		C/G	y	I	H	Hypothetical protein
45	BP3224	9	BB4011	6	BPP3576	nr		C/G	y	C		Energy metabolism, carbon
46	BP3474	13	BB0944	9	BPP0850	7		C/G	y	I	M	Conserved hypothetical
59	BP3624	7	BB0040	7	BPP0040	9		C/G	y	I	H	Degradation of small molecules
61	BP0520	6	BB0444	5	BPP0443	9		C/G	y	I	L	Periplasmic/exported/lipoproteins
62	BP0426	nr	BB0472	7	BPP0472	13	*cspA*	C/G	y	I	L	Adaptation
65			BB1351	9	BPP1135	9		C/G	n	I	H	Global regulatory functions
66	BP1047	7	BB1361	8	BPP1145	10		C/G	y	I	M	Periplasmic/exported/lipoproteins
67	BP1160	8	BB2684	8	BPP1607	11		C/G	y	C		Periplasmic/exported/lipoproteins
71	BP1793	2	BB2270	18	BPP2022	G_12_TG_2_AG_3_		C/G	y	I	L	Cell envelope
**75**	BP1660	G_5_CG_2_CG_3_	BB2741	8	BPP2745	12	*sphB2*	C/G	y	C		Cell envelope
78			BB4881	G_4_ACG_2_	BPP4294	14		C/G	y	I	H	Global regulatory functions
82	BP2738	14	BB2033	35	BPP2591	C_6_TC_5_	*bapC*^*h*^	C/G	y	C		Cell envelope

^a ^HPT number is an arbitrarily assigned unique ID. Table is sorted by order in the *B. bronchiseptica *RB50 genome.

^b ^Identifier of ORF that is either contains or is downstream of the HPT. Empty cell indicates that no orthologous sequence exists in that genome.

^c ^Numbers are length of HPTs. Sequences are given when the tract contains a substitution relative to alleles in the other genomes.

^d ^"y" denotes HPT length polymorphism among the three sequenced genomes.

^e ^"I", located in intergenic region; "C", located in coding sequence

^f ^Probability that the HPT overlaps a promoter coded as follows: H, high; M, moderate; L, low; N, not near a promoter (located in 3' region of convergently transcribed genes); K, known to overlap experimentally defined promoter; U, undetermined. Values were not assigned to coding HPTs. See Methods for definitions of classes.

^g ^Functional categories from MultiFun classification system as assigned by Parkhill, et al. [3]

^h ^Two separate HPTs are found in these genes.

^i ^HPT is upstream of two features by a similar distance.

^j ^"nr" indicates that no HPT is found in the orthologous region.

Among the three species, 51 (74%) of the HPT loci were found to be polymorphic, meaning that the orthologous locus is present in at least two genomes and HPT length differs between them (Table 1). As a result, some of the HPTs in one or two genomes have fewer bases than the selection threshold. Twelve of the HPTs were found in coding regions, with the potential to influence protein length or amino acid sequence. In order to identify tracts that could affect transcriptional regulation, intergenic HPTs were assigned a likelihood of overlapping a promoter, with those located between 21 and 80 nt upstream of the start codon of an ORF being the most likely candidates (Table 1; Additional files 3, 4, and 5). Nineteen HPTs are in this class, including those upstream of the four fimbrial major subunit genes where they have been shown to overlap the functional promoters [26,27]. Although it is more than 80 nt upstream of the start codon, the HPT upstream of *bhuR *was also considered very likely to influence transcription because it maps five bases upstream of the experimentally defined -35 box [32]. In most cases, due to orientation or relative proximity of flanking CDSs, intergenic HPTs could be associated with the regulatory region of a single gene. Intergenic HPTs located in the 3' region of two ORFs or more than 150 nt upstream of the closest ORF start codon are unlikely to be involved in regulation.

Sixteen HPTs were associated with genes encoding cell envelope proteins, and 8 were associated with genes encoding periplasmic or exported proteins, or lipoproteins, for a total of at least 24 genes whose putative products are possible targets for host cell interaction and immune surveillance. Polymorphisms among the three species were more frequent among G/C HPTs (29/30) than among A/T HPTs (21/39) (two-tailed *p *= 0.00007, Fisher's exact test), but this result could be due to the longer tract length of G/C HPTs analyzed here. Due to gene loss in *B. pertussis*, 13 of the *Bordetella *HPT loci identified here are not present in the Tohama I genome. Also, the genes linked to two HPT loci identified in the other species are present in Tohama I, but they are not associated with an orthologous HPT, and two other loci have stabilized HPTs in Tohama I. In summary, we assessed *B. pertussis *to have 36 HPTs that are potentially subject to phase variation, 10 by disrupting an open reading frame, and 26 with high or moderate likelihood of affecting transcriptional regulation. By similar logic, *B. bronchiseptica *and *B. parapertussis *were assessed to have 43 and 42 putative phase-variable HPTs, respectively.

### Identification of *B. pertussis *phase-variable HPTs by PCR/LDR

In order to detect rare tract length alleles in the *B. pertussis *population, and identify variations that may be associated with specific time periods or geographical locations, a large collection of isolates (see below) was assayed for a selected group of HPTs. Six loci were chosen from the list of *Bordetella *HPTs for further investigation of allelic polymorphism (Table 1). Selection of these loci was biased toward HPTs in genes encoding predicted cell envelope proteins (four HPTs) and HPTs that were polymorphic among the three sequenced genomes (five HPTs). Three HPTs – upstream of *fimX *(BP2674), BP0880, and *bhuR *– are predicted to influence transcriptional regulation. HPTs associated with the other three loci – *sphB2 *(BP1660), *tcfA *(BP1201), and *bapC *(BP2738) – are located within the coding sequence. Because HPTs shorter than the threshold lengths used here have been shown to be phase-variable in *B. pertussis *(e.g., *bvgS*), three HPT loci with fewer than 9 C/G base pairs were also chosen for analysis (Table 2). HPTs in BP3651 and BP0059 are polymorphic among the sequenced genomes, and the third, upstream of *ptxA*, is not (Table 2). The HPTs of these nine loci were genotyped by PCR/LDR in a collection of 90 *B. pertussis *strains encompassing a considerable degree of temporal (70 years) and geographic (4 continents) diversity (Additional file 6).

**Table 2:** Additional *B. pertussis*, *B. bronchiseptica*, and *B. parapertussis *HPTs analyzed in this study

	***B. pertussis *Tohama I**		***B. bronchiseptica *RB50**		***B. parapertussis *12822**							
**HPT num**^**a**^	**ORF**^**b**^	**Allele**^c^	**ORF**	**Allele**	**ORF**	**Allele**	**Gene**	**Base**	**Poly-morphic**^**d**^	**Loc**^**e**^	**Promoter overlap**^**f**^	**Functional category**^**f**^

**47**	BP0059	8	BB0530	6	BPP0525	6		C/G	y	C		Global regulatory functions
**81**	BP1877	6	BB2995	6	BPP3029	6	*bvgS*	C/G	n	C		Global regulatory functions
**57**	BP3651	8	BB0066	6	BPP0066	6		C/G	y	I	H	Cell envelope
**85**	BP3783	6	BB4890	6	BPP4304	6	*ptxA*	C/G	n	I	K	Periplasmic/exported/lipoproteins

^a ^HPT number is an arbitrarily assigned unique ID. Table is sorted by order in the *B. bronchiseptica *RB50 genome.

^b ^Identifier of ORF that is either contains or is downstream of the HPT. Empty cell indicates that no orthologous sequence exists in that genome.

^c ^Numbers are length of HPTs. Sequences are given when the tract contains a substitution relative to alleles in the other genomes.

^d ^"y" denotes HPT length polymorphism among the three sequenced genomes.

^e ^"I", located in intergenic region; "C", located in coding sequence

^f ^Probability that the HPT overlaps a promoter coded as follows: H, high; K, known to overlap experimentally defined promoter.

Values were determined only for coding HPTs. See Methods for definitions of classes.

^g ^Functional categories from MultiFun classification system as assigned by Parkhill, et al. [3]

If Tohama I were a representative *B. pertussis *strain, variant alleles of each HPT in other strains would be most often either one base pair longer or shorter than the Tohama I allele. Thus, for each locus, three discriminating oligonucleotides were synthesized: one each targeting the HPT in the Tohama I allele, the -1 allele (relative to Tohama I), and the +1 allele (Additional file 7). The BP3651, *fimX*, BP0880, BP0059 and *sphB2 *HPTs were screened with uniplex reactions that contained the single discriminating oligonucleotide that targets the Tohama I allele, and the FAM-labeled common oligonucleotide. The remaining HPTs were screened by multiplex PCR/LDR reactions in which all three discriminating oligonucleotides were mixed with a FAM-labeled common oligonucleotide. Strains yielding PCR/LDR profiles different from Tohama I were further analyzed by sequencing the PCR product containing the variable HPT. In cases where no ligation products were detected, HPT length was determined by sequencing.

**Figure 3:**
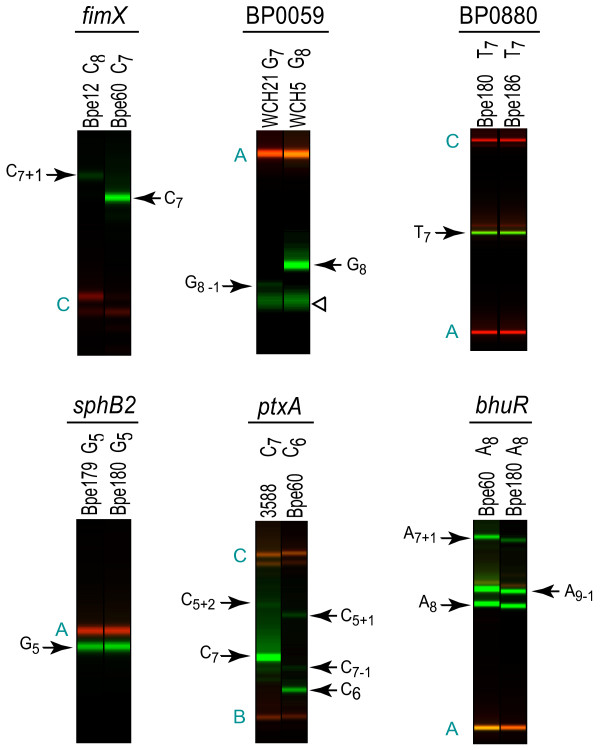
**Representative PCR/LDR results for HPTs in *fimX*, BP0059, BP0880, *sphB2*, *ptxA*, and *bhuR***. Raw capillary electrophoresis data for ligation products (green) and molecular weight standards (red) displayed as if an electrophoretic gel image. Assayed locus is indicated at the top of each panel; strain and length of its HPT allele are noted above each lane. Representative LDR results are depicted here; for complete data from all strains see Additional files 8, 9, 10, 11, 12, and 13. BP0880, BP0059, *fimX*, and *sphB2 *HPTs were screened by uniplex LDR reactions containing only the discriminating oligonucleotide that targets the Tohama I sequence. The *ptxA *and *bhuR *HPTs were screened by multiplex reactions that contain all three discriminating oligonucleotides. Letters in teal indicate molecular weight standards as labeled in the corresponding supplementary figures. Ligation products (arrows) are labeled using the notation described in the legend to Figure 1. The open arrowhead indicates a synthesis artifact in the BP0059 common oligonucleotide preparation.

Among the strains examined, the HPTs in BP0880, *sphB2*, and *bhuR *were not polymorphic, indicating that they are stable in the *B. pertussis *population (Figure 3; Additional files 8, 9, and 10). Two HPTs varied in only a single *B. pertussis *strain. The coding HPT in BP0059 contained one additional base in WCH21, an isolate from Australia (Figure 3 and Additional file 11). This HPT is the site of a frameshift mutation that truncates the ORF in *B. pertussis *Tohama I. The single base insertion in WCH21 restores the reading frame and is predicted to result in a full-length protein; all other strains assayed carry the frameshifted allele. The HPT upstream of *ptxA *varied by the addition of a single base in strain 03588, isolated in Italy in 1993 (Figure 3 and Additional file 12). This insertion increases the distance between the predicted -35 and -10 boxes of the *ptx-ptl *operon [33]. Changing the distance between these regulatory elements by as few as two bases has been shown to reduce dramatically transcription of this key toxin expression locus, suggesting that the HPT variation in 03588 may have significant consequences for expression [34].

The five HPTs described above maintained a constant length over the duration of each *B. pertussis *batch culture experiment (representing 10^9 ^bacteria). Since the detection limit of the assay is 1 variant allele in 100, the proportion of mutants after 30 [log_2_(10^9^)] generations has an upper bound of 0.01. Using the mathematical model developed by Saunders, *et al*. [35], the maximum phase variation rate for these loci, assuming equal forward and back mutation rate and no fitness difference, is 6.7 × 10^-4 ^per generation.

### HPT variation in *fimX *and other fimbrial major subunit gene promoters

In seven strains, the HPT upstream of *fimX *differed from the Tohama I allele (C_7_) by the addition of a single base (Figure 3; Additional files 13 and 14). After excluding replicate isolates, four independent strains with the longer allele remained. This HPT is also known as the "C-stretch" motif of a previously identified fimbrial gene promoter element, which also contains the conserved "fim box" [26,27]. The length of the C-stretch in the *fim2 *and *fim3 *promoters has been shown to influence transcriptional activity [26,27]. Furthermore, deliberate mutation of the C-stretch upstream of *fimX *affected transcription of that gene [26]. In order to examine whether *fimX *expression differed between the allelic variants in our collection, quantitative RT-PCR was used to assay *fimX *transcript abundance in a sample of strains harboring C_8 _versus C_7 _tracts. Linear regression analysis showed a strong correlation (*R*^2 ^= 0.611) between transcript abundance and tract length, in which strains with the C_8 _HPT had an average *fimX *transcript abundance 3.8-fold lower than those with a C_7 _HPT (*p *= 0.0137) (Additional file 14). Although this result strongly suggested that a single base length polymorphism in this HPT may influence *fimX *expression, differential effects due to cis- or trans-regulatory factors cannot be ruled out. However, the sequences of the 64 bases upstream of the C-stretch and 87 bases downstream, including the translational start site, did not differ between the strains examined. Among the 51 independent clinical isolates from the United States and the Netherlands for which vaccine history was known, all three of the C_8 _*fimX *HPT alleles were from strains isolated prior to the introduction of vaccination (1949 in the United States, and 1953 in the Netherlands; two-tailed *p *= 0.0146, Fisher's exact test).

Other *B. pertussis *and *B. bronchiseptica *fimbrial major subunit genes exhibit allelic polymorphisms in coding regions [36-38] and in the long cytosine HPTs (up to C_19 _in *B. bronchiseptica fim3*) in their promoters [26,27,39]. Because the data above suggest a shift in *fimX *HPT alleles subsequent to vaccine introduction, the HPTs upstream of *fim2 *and *fim3 *were assayed for evidence of a concomitant change. Because HPTs greater than 16 nt are not reliably measured by LDR [28], these HPTs were characterized by direct sequencing of PCR products, recognizing that the generation of slippage products in the PCR and sequencing reactions remains a potential source of experimental error.

HPT lengths for *fim2 *ranged in this set of *B. pertussis *strains from 10 to 15. As with *fimX *HPT length polymorphisms, the pre-vaccine strains (median = 13.5) had significantly longer *fim2 *HPTs than post-vaccine strains (median = 11; *p *= 0.0339, Mann-Whitney U-test; Additional file 14). Likewise, HPT lengths for *fim3 *ranged in this sample from 13 to 18; pre-vaccine strains (median = 14.5) tended to have longer tracts than post-vaccine strains (median = 13), but this difference did not achieve statistical significance (*p *= 0.0771, Mann-Whitney U-test).

### HPTs that varied during a single round of culture

**Figure 4:**
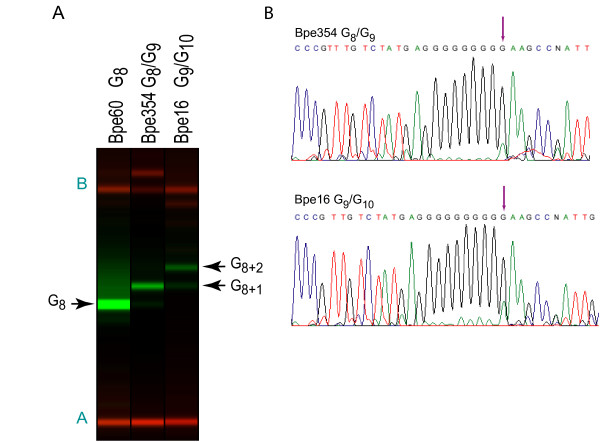
**Representative PCR/LDR data for the BP3651 HPT**. (**A**) Raw capillary electrophoresis data for ligation products from BP3651 uniplex PCR/LDR (green) and molecular weight standards (red) displayed as if an electrophoretic gel image. Strains are indicated above each lane. Ligation products (arrows) are labeled using the notation described in the legend to Figure 1. Letters in teal indicate molecular weight standards as labeled in Additional file 16. (**B**) Partial traces from direct sequencing of BP3651 PCR products. Strain and alleles are noted above each sequence trace. Purple arrows indicate the point in the sequence trace after which peak shadowing, indicative of a mixture of HPT alleles, can be observed.

The HPTs of *tcfA*, *bapC*, and BP3651 exhibited tract length variation between strains, and within genomic DNA samples derived from cultures of 10^9 ^bacteria grown from single colonies over 30 [log_2_(10^9^)] generations (Additional file 15). Variant alleles of the HPT upstream of BP3651 were found in six strains (Figure 4; Additional files 15 and 16). The atypical *B. pertussis *strain, 18323, represented by three separate isolates, harbored a mixture of G_9 _and G_10 _alleles. Three other independently isolated strains displayed a mixture of G_8 _and G_9 _alleles. In all other strains in this collection, only the G_8 _allele was detected, suggesting that this HPT may be unstable only when its length exceeds a critical threshold of 8 bases. In order to determine whether allelic variation was present in smaller populations founded by a single cell, five independent single colonies (approximately 10^7 ^bacteria; data not shown) of two variable strains, Bpe354 and Bpe16, were assayed by colony PCR/LDR. In all cases, each of the single colonies yielded mixed LDR profiles, indicating that allelic variation in the BP3651 HPT arises over the approximately 23 [log_2_(10^7^)] generations required to produce a macroscopic colony.

**Figure 6:**
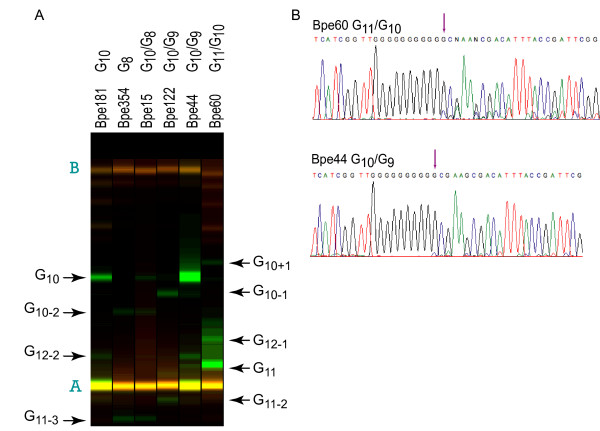
**Representative PCR/LDR data for the *bapC *HPT**. Raw capillary electrophoresis data for ligation products from *bapC *multiplex PCR/LDR (green) and molecular weight standards (red) displayed as if an electrophoretic gel image. Strains and HPT allele lengths are indicated above each lane. Ligation products (arrows) are labeled using the notation described in the legend to Figure 1. Letters in teal indicate molecular weight standards as labeled in Additional file 18. (**B**) Representative traces from direct sequencing of *bapC *PCR products. Strain and alleles are noted above each sequence trace. Purple arrows indicate the point in the sequence trace after which peak shadowing, indicative of a mixture of HPT alleles, can be observed.

The predominant *bapC *HPT allele in 76/90 isolates was G_11_, but almost all of these also harbored a detectable fraction of G_10 _and G_12 _alleles (Figure 6 and Additional file 18). Six strains carried primarily the G_10 _allele, two carried primarily the G_9 _allele, and four carried primarily the G_8 _allele, but in most of these cases, a mixture of alleles was detected. In many cases, mixed allelic content was verified by sequencing (Figure 6). In order to assess allelic variation in small populations founded by a single cell, 93 independent Bpe60 colonies, each containing approximately 1 × 10^7 ^bacteria, were assayed by colony PCR/LDR. In every case, multiple alleles were detected, with the G_11 _allele predominating, but G_10_, G_12_, and in some cases G_13 _alleles also present (Additional file 19), indicating that allelic variation in the *bapC *HPT also arises within 23 generations. The G_11 _tract, as annotated in the Tohama I genome sequence, causes a shift in the reading frame that is predicted to result in a truncated BapC protein. However, a single base contraction of the HPT would restore the reading frame, leading to synthesis of a full-length protein.

**Figure 5:**
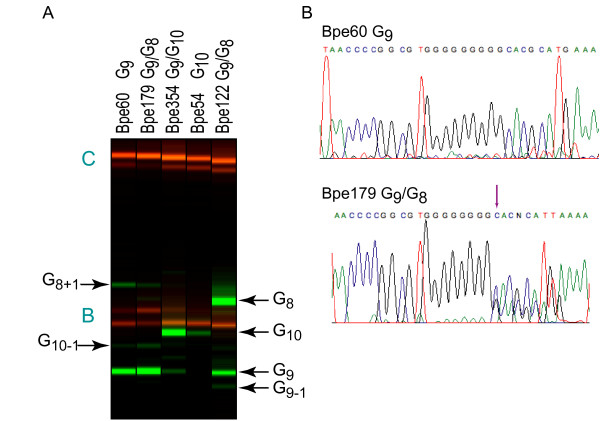
**Representative PCR/LDR data for the *tcfA *HPT**. (**A**) Raw capillary electrophoresis data for ligation products from *tcfA *multiplex PCR/LDR (green) and molecular weight standards (red) displayed as if an electrophoretic gel image. Strains are indicated above the lane. Ligation products (arrows) are labeled using the notation described in the legend to Figure 1. Letters in teal indicate molecular weight standards as labeled in Additional file 17. (**B**) Representative traces from direct sequencing of *tcfA *PCR products. Strain and alleles are noted above each sequence trace. Purple arrow indicates the point in the sequence trace after which peak shadowing, indicative of a mixture of HPT alleles, can be observed.

The predominant *tcfA *allele in 82/90 strains was G_9_, but the G_8 _allele was detected as a minor allele in 75 of these (Figure 5 and Additional file 17). One strain, Bpe122, yielded approximately equal numbers of G_8 _and G_9 _alleles. Five strains carried primarily the G_10 _allele, but four of these also harbored a detectable fraction of G_9 _and G_11 _alleles. One- or two-base insertions into the G_9 _HPT, beginning at codon 130 of the *tcfA *ORF, alter the reading frame, and presumably result in a truncated, non-functional Tcf protein. Stability of the *tcfA *HPT was further assessed by cloning PCR products containing the HPT into a plasmid in *E. coli*. Eight independent *E. coli *clones harboring PCR products from the Bpe354 genomic DNA preparation, which carried predominantly G_10 _alleles, but detectable G_9 _alleles, were assayed by colony PCR/LDR. Five of the clones carried a mixture of alleles similar to the original Bpe354 genomic DNA preparation, while three clones carried a mixture of G_10 _and G_11 _alleles, indicating that allelic contraction and expansion at this locus occurs in *E. coli *(data not shown).

## Discussion

*B. pertussis *is unusual among bacterial pathogens in its limited genomic diversity. Multiple methods, including MLST, MLEE, and CGH, have found that *B. pertussis *is highly clonal with very few genomic differences, even among temporally and geographically diverse strains [4,10,11]. Phase variation could be an important mechanism in *B. pertussis *for generation of phenotypic diversity in the absence of genome plasticity. In fact, prior to this study, a small number of key virulence phenotypes of *B. pertussis *were already known to be phase-variable.

This study focused on the identification and characterization of HPTs, because they are prone to expansion and contraction and often associated with bacterial phase-variable loci. Evidence of phase variation can be obtained by demonstrating repeat length polymorphism within multiple genomes from the same species, or closely related species as in this study. Scanning multiple related genomes has the added benefit of identifying polymorphic, putative, phase-variable loci that fail to meet the identification threshold in one strain but meet it in another. For example, if only the TohamaI genome had been scanned, 40 HPTs would have been identified,; however, the comparative approach identified 12 more HPTs that are shorter than the threshold in *B. pertussis *but meet the threshold in at least one of the closely related *Bordetella *genomes.

The comparative approach, while identifying HPTs that have changed at least once since the divergence of the two genomes, does not address the key issue of allelic variation rate. Are these differences stable over the long time periods that differentiate bacterial lineages, or do they fluctuate rapidly, with each genome sequence providing only a snapshot of the genome at a specific point in time? An estimate of HPT variation frequency can be obtained by measuring repeat length in multiple genomes, and doing so with a method sensitive enough to detect rare variants in a mixture of alleles. PCR/LDR is an ideal method for detecting rare genotypes as it can clearly delineate numerous genotypes within a mixed sample. While the assay is not quantitative, its sensitivity allows detection of one variant HPT in 100 ([28]; this study). With a sequencing-based approach, assuming a Poisson distribution, 300 clones would need to be analyzed to have a 99% chance of detecting a variant allele representing 1% of the sample.

The frequency of allelic variation differed greatly among the nine HPTs genotyped in this study. Three HPTs were invariant among the 90 *B. pertussis *isolates, two differed in a single strain, and one differed between strains isolated during different eras. Because no single genomic DNA preparation harbored a detectable mixture of HPT alleles, a maximum mutation rate of 6.7 × 10^-4 ^mutations per generation was inferred for these six loci. The actual mutation rate could be significantly lower, but accurate determination would require identification of cultures containing variant alleles, and estimation of the relative fitness of each genotype, both of which are impractical due to the low apparent rate of variation and lack of a known selectable phenotype. Three HPTs exhibited allelic polymorphism within single colonies. The HPT in BP3651 was variable within a single culture only in strains with alleles longer than C_8_, leading us to speculate that an HPT might become relatively unstable with the addition of a single base. The mechanisms that regulate the frequency of HPT phase variation are not clearly understood, but conditions that influence the activity of the mismatch repair pathway have been shown to affect the frequency of phase variation [40]. Although the mutation rate at the BP3651, *tcfA*, and *bapC *HPTs is apparently much higher than for the other HPTs examined here, accurate determination of these rates would require quantitative estimates of the proportion of each allele, which is not possible using LDR. The relative fitness of each genotype is also a key parameter in this calculation [35], but because stable genotypes cannot be isolated in culture, these measurements cannot easily be made. However, fitness differences would reduce frequency of deleterious alleles, making the mutation rate appear lower than it actually is.

The discovery here of a *B. pertussis *strain with an expanded HPT in a region of the *ptx *promoter known from mutational studies to be critical for transcriptional activity [34] suggests that *ptxA *expression may be atypical in this strain. Although numerous studies have documented allelic variation in the coding region of *ptxA *(e.g., [36,41,42]), only one study focused on potential regulatory polymorphisms in the *ptx *promoter region, identifying rare single nucleotide polymorphisms further upstream of *ptxA*, but not in the HPT [43]. Allelic shifts subsequent to the introduction of vaccination in the *ptxA *coding region support a model of vaccine-driven evolution [44]. Perhaps modified expression of PT could also confer a fitness advantage in a vaccinated host population. These possibilities deserve and require further investigation.

Two genes encoding autotransporter family proteins had the most highly variable HPTs among the loci assayed. The *tcfA *gene encodes tracheal colonization factor (Tcf), a secreted protein produced by *B. pertussis *[45]. Disruption of this gene resulted in a 10-fold reduction in colonization of a mouse model, suggesting that it is an important virulence determinant [45]. The phase-variable HPT is located in the *tcfA *open reading frame such that expansions and contractions of the tract would result in the reversible truncation, and presumed inactivation, of the Tcf protein product. This HPT is the location of the frameshift polymorphism previously identified in the *tcfA5 *allele, which was proposed to represent a *tcfA *phase variant [41]. The PCR/LDR results suggest that, within a culture of *B. pertussis*, only a fraction of cells express full-length TcfA. The prevalence of antibodies against TcfA in human populations has not been addressed, but the existence in the *B. pertussis *population of multiple *tcfA *alleles that encode different protein variants suggests that it may be under selection by immune surveillance [41]. If so, *B. pertussis *cells that express Tcf *in viv*o might be more rapidly cleared by the immune system, and cells that do not express Tcf or express a variant form, as a result of phase variation, would have a selective advantage. However, through the continuous emergence of Tcf-expressing phase variants within the population, production of this secreted virulence factor could be maintained in temporal phases or niches where the benefits of expressing it outweigh the risk of increased susceptibility to immune clearance.

Frequent phase variation was also observed at the HPT in the coding region of the *bapC *gene. By analogy to other *B. pertussis *autotransporter family proteins that have been implicated in pathogenesis, a role for this gene in virulence has been proposed [46], but such a function has not yet been demonstrated. Likewise, nothing is known about the immune response to BapC, but its predicted localization on the cell surface makes it a plausible target for immune surveillance. In a previous study, partial sequencing of *bapC *from 10 *B. pertussis *strains identified only a silent SNP [46], but the 5' end of the gene, which contains the HPT, was not examined. Interestingly, *bapC *harbors a second HPT (C_14 _in Tohama I), upstream of the HPT assayed here, that may also be phase-variable (Table 1).

By characterizing *fimX *allelic polymorphism and gene expression, this study expands upon previous work on phase variation of fimbrial major subunit genes. Among the strains examined, seven were found to harbor a C_8 _HPT in the *fimX *promoter region, distinguishing them from the majority of strains which carried a C_7 _tract. *fimX *transcript abundance was found to be nearly fourfold higher in C_7 _strains than in C_8 _strains, suggesting that this single base difference may significantly influence transcription, and arguing against the model in which fimbrial gene expression is only activated when the distance between the activator binding region and the -10 box exceeds a threshold of 22 bases [26,27]. The data presented here suggest that the activity of these promoters may be more subtly regulated by HPT expansions and contractions, even when the tract is relatively short.

The C_8 _allele of the HPT in the *fimX *promoter was found only in *B. pertussis *strains isolated prior to the introduction of pertussis vaccines, suggesting that vaccination may have led to selection against strains carrying this allele. Because the C_8 _HPT correlates with decreased *fimX *transcript levels, we propose that increased FimX expression may confer a selective advantage in an immunized host population. In *B. pertussis *populations from multiple countries, initiation of a vaccination program using a Fim2-expressing vaccine strain has led to the significant enrichment of Fim3-expressing isolates (reviewed in [38]. By analogy, vaccine-induced selection against Fim2 may have also led to the enrichment of strains that express more FimX. Such a shift would not have been detected by serotyping because only Fim2 and Fim3 antisera have been used in these assays. The substitution of one fimbrial subtype for another has been proposed as a mechanism to evade the immune system without substantial loss of adhesion [47].

## Conclusion

Computational screening was used to identify significantly long HPTs that were predicted to be prone to length polymorphism in the genomes of three closely related *Bordetella *species. A comparative genomic approach verified that many of these loci are, in fact, polymorphic among the three species. In order to obtain evidence for HPT length polymorphism within a single species, nine HPT loci were genotyped using the PCR/LDR assay in a collection of 90 *B. pertussis *strains. Polymorphism was observed at six of these loci, suggesting that a thorough examination of all 52 *B. pertussis *HPTs could reveal dozens of previously unrecognized phase-variable genes. Three HPTs displayed tract length polymorphism within cultures derived from single colonies, indicating that they are rapidly phase-variable. A fourth polymorphic HPT was identified in the promoter of *fimX *where a single base expansion variant was present only among strains that were isolated prior to introduction of pertussis vaccines. Transcript abundance of *fimX *was found to be 3.8-fold lower in strains carrying the longer allele.

Given the diversity of alternate phenotypes that may be generated by the 69 HPTs identified in this study, it is likely that phase variation is involved in many aspects of *Bordetella *pathogenesis. Because *B. pertussis *lacks genetic diversity, phase variation may be a key mechanism for adaptation to the hostile and changing host environment. Future work to elucidate functional differences associated with the phase variants described in this study may lead to new insights into *Bordetella *host specificity, immune system modulation, and niche adaptation.

## Methods

### Strains, media, and genomic DNA preparation

Strains used in this study are described in Additional file 6. Pre-vaccination era strains in the U.S. were defined as strains isolated before 1949, when use of the whole-cell pertussis vaccine became widespread [48]. U.S. strains isolated subsequent to 1949 were considered to be post-vaccination era strains. Strains from the Netherlands were considered pre-vaccination era strains if isolated prior to 1953, and post-vaccination era strains if isolated after 1953 [37]. Strains from Australia and Italy were not included in the statistical analysis of *fimX *polymorphisms, as pre- and post-vaccination status for each strain was not available.

Single *B. pertussis *colonies from Bordet-Gengou agar (BD, Franklin Lakes, New Jersey, United States) plates supplemented with 15% defibrinated sheep blood (BG blood plates) were inoculated into modified Stainer-Scholte media (SS) and grown to stationary phase. Genomic DNA was purified from bacterial cell pellets using the Wizard Genomic DNA Purification Kit (Promega, Madison, WI, USA) as previously described [2].

### Homopolymeric tract identification and statistical analysis

HPTs were identified by scanning *Bordetella *genome sequences (RefSeq NC_002927, NC_002928, and NC_002929) using the equicktandem program of the EMBOSS package [49]. Markov model transition probabilities and observed number of HPTs were calculated using calc_transition_matrices.pl and count_HPTs.pl (available at [50]). For C or G HPTs, second through fifth order Markov models yielded very similar observed/expected curves, and for A or T HPTs, first and second order Markov models yielded very similar observed/expected curves, but higher order Markov models may exhibit overfitting. Orthologous HPTs in the three *Bordetella *genomes were manually confirmed using ACT [51], and alignments were verified using cross_match [52] and ClustalW [53]. Routine statistical analyses were performed using StatView v. 5.0.1 software (SAS Institute, Cary, NC, USA).

The position of each intergenic HPTs was mapped relative to adjacent genes in order to determine whether it could influence transcriptional regulation. Because most *Bordetella *promoters have not been functionally characterized, we made the assumption that the distribution of distances between promoter and start codon would follow a similar trend to the distribution found in the *E. coli *genome [54]. Extrapolating from this distribution, HPTs upstream of at least one gene were considered highly likely to overlap a promoter element when located between 21 and 80 nt upstream of the start codon of an ORF. HPTs located fewer than 21 nt, or between 81 and 150 nt upstream of the start codon were considered to have a moderate likelihood of overlapping a promoter, while those more than 150 nt upstream were considered to have a low probability. HPTs between adjacent, convergently transcribed ORFs were assumed not to overlap a promoter.

### PCR, cloning and sequencing

All PCR amplifications, unless otherwise indicated, employed *Pfu *Ultra or *Pfu *Turbo High Fidelity DNA polymerase (Stratagene, La Jolla, CA, USA) according to the manufacturer's instructions. Fidelity comparison experiments used AmpliTaq DNA Polymerase (Ambion, Austin, TX, USA). 25 μl reaction volumes contained 25 ng of genomic DNA, 10 mM dNTPs, and 20 pmol of each oligonucleotide primer (see Additional file 20 for PCR primers used). PCR parameters were 5 min at 96°C; followed by 30 cycles of 95°C for 30 s, 55°C for 30 s, 72°C for 1 min; and a final 7 min extension step at 72°C. Reactions were then heated for 30 minutes at 96°C to inactivate the polymerase. 5 μl of the PCR reaction was visualized on a 2% agarose gel to confirm the presence of an amplification product of the expected size. Selected PCR products were sequenced directly (Elim Biopharmaceuticals, Hayward, CA, USA) using one amplification primer. PCR oligonucleotide primer (Integrated DNA Technologies, Coralville, IA, USA) sequences are in Additional file 20.

Some PCR products were cloned as blunt-ended molecules using the Zero Blunt TOPO PCR Cloning Kit and the pCR II-Blunt TOPO vector (Invitrogen, Carlsbad, CA, USA), according to the manufacturer's instructions, with the following modifications to maximize efficiency when cloning a pool of PCR products: 4 μl of PCR product, 1 μl Salt Solution and 1 μl vector were incubated for 20 minutes; 4 μl of the cloning reaction was added to a vial of One Shot Chemically Competent *E. coli*, which was then gently mixed and incubated on ice for 5 minutes.

Colony PCR of *B. pertussis *strains was performed on pinprick size colonies grown on BG blood plates at 37°C by resuspending a single colony in 10 μl of 1× *Pfu *Ultra High Fidelity Polymerase Buffer (New England BioLabs, Ipswitch, MA, USA), and incubating at 96°C for 10 minutes. The resulting cell lysate was centrifuged at 600 × *g *for 1 min. 2 μl of the cleared lysate was used as the genomic template for subsequent PCR reactions as described above.

### LDR conditions

Uniplex PCR/LDR reactions contained 1 μl of a PCR reaction, 25 nmol of labeled common oligonucleotide, and 25 nmol of one unlabeled discriminating oligonucleotide. Multiplex PCR/LDR reactions contained 1 μl of PCR reaction mix, 25 nmol of labeled common oligonucleotide, and 25 nmol each of three different unlabeled discriminating oligonucleotides. All LDR oligonucleotides were synthesized and HPLC purified by Integrated DNA Technologies (see Additional file 7). All PCR/LDR reactions were performed in 1× *Tth *DNA Ligase Buffer (New England BioLabs) at a final volume of 20 μl. The reaction mixture was heated for 1.5 min at 94°C prior to adding 25 fmol of *Tth *DNA Ligase (New England BioLabs). LDR reactions were cycled 30 times, each cycle consisting of 15 s at 94°C and 2 min at 65°C. Reactions were stopped with 1.5 μl of 0.5 mM EDTA, then spiked with a custom mixture of fluorescent 2',7'-dimethoxy-4',5'-dichloro-6-carboxyfluorescein (JOE)-labeled oligonucleotide molecular weight standards (Integrated DNA Technologies) at 100 fmol. Reactions were analyzed using an ABI 3100 capillary sequencing instrument (Applied Biosystems, Foster City, CA, USA) at the Stanford Protein and Nucleic Acid Biotechnology Facility (Stanford, CA, USA). In cases where no ligation product was detectable, PCR products were sequenced directly to determine HPT length.

### LDR data analysis

Raw trace data from ABI format output files were displayed as images using gel_draw.pl. Molecular weights of ligation products were calculated from raw trace data by comparing their mobility to that of oligonucleotide standards using ABImulti.py. Both of these scripts utilize the convert_trace and scf_dump programs of the Staden package [55] for extraction of raw fluorescence intensity data from ABI format output files. Software described in this manuscript is available for download at [50].

### Measurements of gene expression

Cultures of *B. pertussis *were established by inoculating a single colony from a BG blood plate into 1.5 ml of SS, incubating at 37°C with shaking until an OD_600 _of approximately 2 was achieved, and sub-cultured, in duplicate, to an OD_600 _of 0.05 in SS. These duplicate cultures were then incubated at 37°C with shaking until aliquots were harvested for RNA extraction during mid-log phase (OD_600 _1.5 – 2.5). RNA was extracted and reverse transcribed as previously described [56]. Quantitative RT-PCR reactions were carried out in a final volume of 20 μl using IQ Sybrgreen Supermix 2× Mix for Real-time PCR (Bio-Rad Laboratories, Hercules, CA, USA), 1 μl cDNA (in RT reaction mix), 1 μl DMSO, and 6 pmol each forward and reverse primers (Integrated DNA Technologies; see Additional file 20). Real-time PCR was performed with an ABI Prism 7900 HT sequence detection system (Applied Biosystems). Data were analyzed using the ΔΔC_t _method with *recA *as the reference [57].

## Abbreviations

PCR: polymerase chain reaction

LDR: ligase detection reaction

HPT: homopolymeric tract

PT: pertussis toxin

JOE: 2',7'-dimethoxy-4',5'-dichloro-6-carboxyfluorescein

FAM: 5-carboxyfluorescein

## Authors' contributions

EBG designed, performed, and analyzed molecular assays, and drafted the manuscript. CAC conceived of the study, participated in its design, performed bioinformatics and statistical data analysis, developed LDR visualization software, and drafted the manuscript. RCB developed software tools for LDR data analysis. DAR helped in the design of the study, guided its execution, and contributed to interpretation of the data, and preparation of the manuscript. All authors read and approved the final manuscript.

## Supplementary Material

Additional file 1 Supplementary Figure 1. Capillary electrophoresis traces of HPLC-purified *bvgS *LDR oligonucleotides. Click here for file

Additional file 2 Supplementary Figure 2. Fidelity of Pfu and Taq DNA polymerases over multiple rounds of PCR/LDR. Click here for file

Additional file 3 Supplementary Table 1. Characteristics of *B. bronchiseptica *HPTs Click here for file

Additional file 4 Supplementary Table 2. Characteristics of *B. pertussis *HPTs Click here for file

Additional file 5 Supplementary Table 3. Characteristics of *B. parapertussis *HPTs Click here for file

Additional file 6 Supplementary Table 4. Characteristics of *B. pertussis *strains used in this study Click here for file

Additional file 7 Supplementary Table 5. LDR and molecular weight standard oligonucleotides Click here for file

Additional file 8 Supplementary Figure 3. BP0880 PCR/LDR. Click here for file

Additional file 9 Supplementary Figure 4. *sphB2 *PCR/LDR. Click here for file

Additional file 10 Supplementary Figure 5. *bhuR *PCR/LDR. Click here for file

Additional file 11 Supplementary Figure 6. BP0059 PCR/LDR. Click here for file

Additional file 12 Supplementary Figure 7. *ptxA *PCR/LDR. Click here for file

Additional file 13 Supplementary Figure 8. *fimX *PCR/LDR. Click here for file

Additional file 14 Supplementary Table 6. Lengths of HPTs in fim gene promoters and relative expression of *fimX *transcript. Click here for file

Additional file 15 Supplementary Table 7. Lengths of HPT alleles detected by PCR/LDR for three hypervariable HPT loci. Click here for file

Additional file 16 Supplementary Figure 9. BP3651 PCR/LDR. Click here for file

Additional file 17 Supplementary Figure 10. *tcfA *PCR/LDR. Click here for file

Additional file 18 Supplementary Figure 11. *bapC *PCR/LDR. Click here for file

Additional file 19 Supplementary Figure 12. Bpe60 colonies screened for *bapC *HPT length Click here for file

Additional file 20 Supplementary Table 8. PCR and RT-PCR oligonucleotide primers. Click here for file
