# Supplementary material for: Phase variation and microevolution at homopolymeric tracts in Bordetella pertussis
Source: BMC Genomics. 2007 May 17;8:122. doi: 10.1186/1471-2164-8-122 (PMC1891110; doi:10.1186/1471-2164-8-122)
Supplement: Additional file 1 — Supplementary Figure 1. Capillary electrophoresis traces of HPLC-purified bvgS LDR oligonucleotides. [file 1471-2164-8-122-S1.pdf]

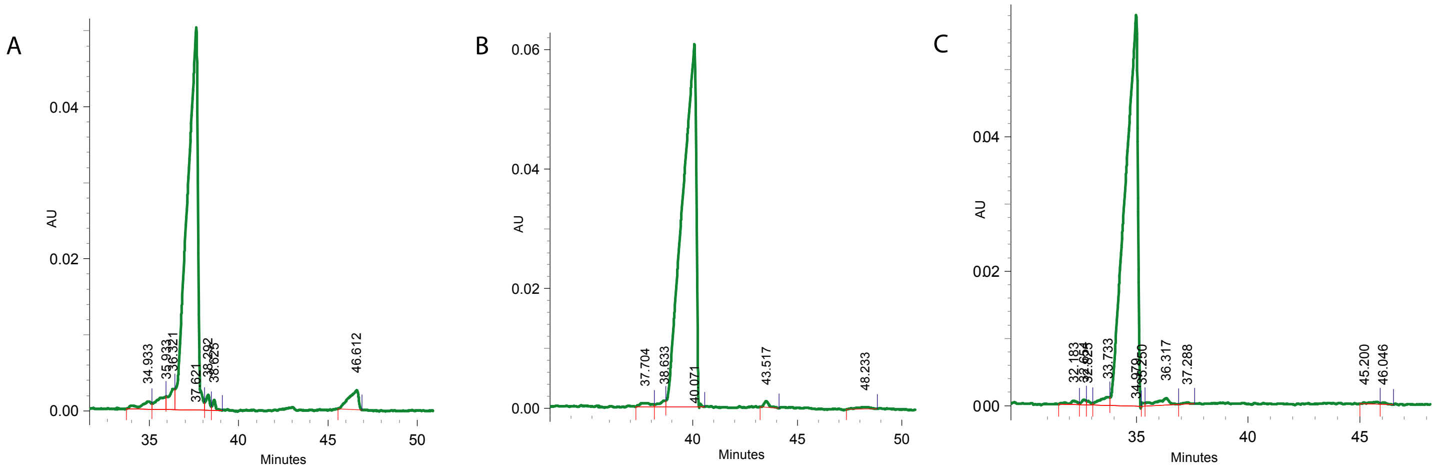

Supplemental Figure 1. Capillary electrophoresis traces of HPLC-purified bvgS LDR oligonucleotides
HPLC-purified oligonucleotides were assayed for purity by capillary electrophoresis by the manufacturer (IDT). Elution time in minutes is plotted on the X-axis and absorbance units at 254 nm (AU) are plotted on the Y-axis. (A) bvgS-common, (B) bvgS-LDR-C7, (C) bvgS-LDR-C6.
