# Supplementary material for: Phase variation and microevolution at homopolymeric tracts in Bordetella pertussis
Source: BMC Genomics. 2007 May 17;8:122. doi: 10.1186/1471-2164-8-122 (PMC1891110; doi:10.1186/1471-2164-8-122)
Supplement: Additional file 2 — Supplementary Figure 2. Fidelity of Pfu and Taq DNA polymerases over multiple rounds of PCR/LDR. [file 1471-2164-8-122-S2.pdf]

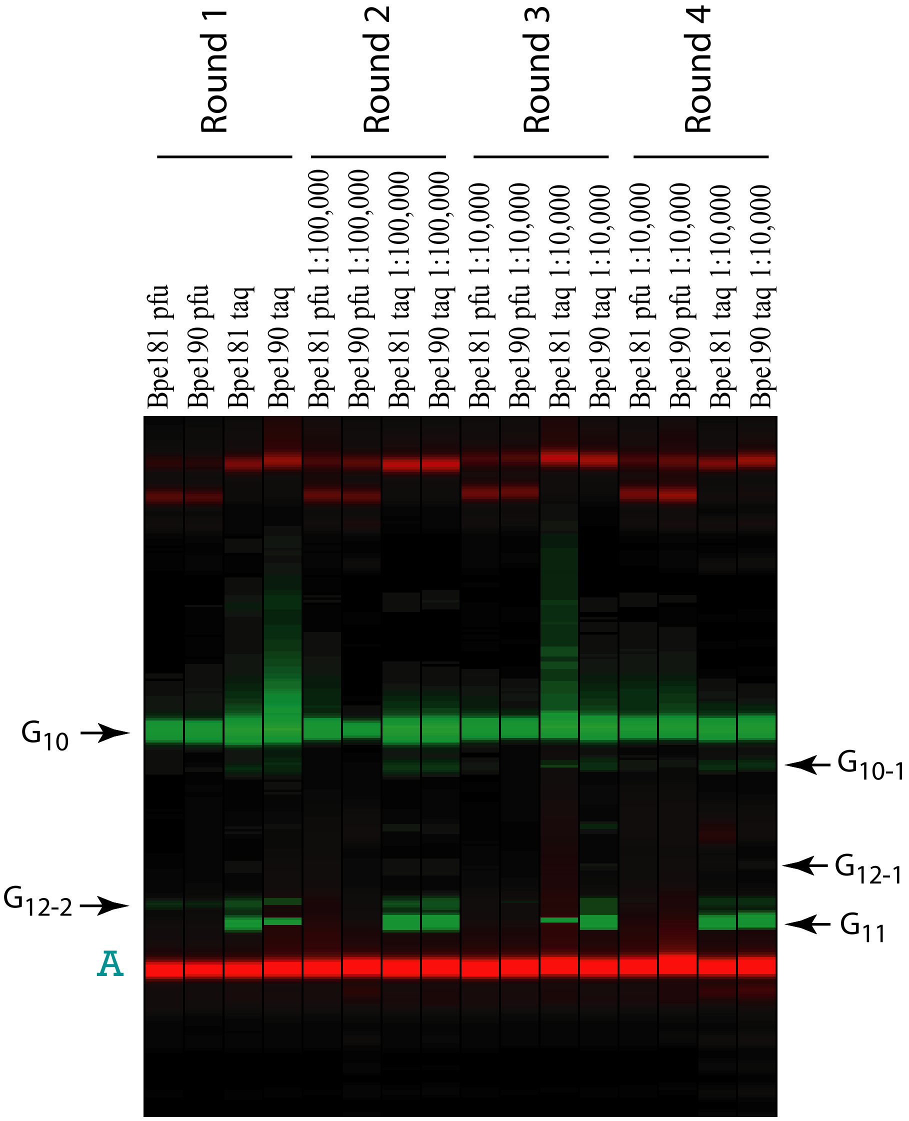

Supplementary Figure 2. Fidelity of Pfu and Taq DNA polymerases over multiple rounds of PCR/LDR. Raw capillary electrophoresis data for ligation products from *bapC* multiplex PCR/LDR (green) and molecular weight standards (red) displayed as if an electrophoretic gel image, with higher molecular weight oligonucleotides closer to the top of the image. Each of two genomic DNA templates (Bpe181 and Bpe190) was amplified with either Pfu Ultra High Fidelity DNA Polymerase (Pfu) or AmpliTaq DNA Polymerase (Taq) then assayed by LDR. PCR rounds (30 cycles each) are indicated across the top of the figure.

In Round 1, 25 ng of template was used. In subsequent rounds, a dilution (indicated above each lane) of the previous round reaction served as template for PCR. Ligation products (arrows) are labeled using the notation described in the legend to Figure 1. Letters in teal indicate molecular weight standards as labeled in Supplementary Figure 12.
