# Supplementary material for: Phase variation and microevolution at homopolymeric tracts in Bordetella pertussis
Source: BMC Genomics. 2007 May 17;8:122. doi: 10.1186/1471-2164-8-122 (PMC1891110; doi:10.1186/1471-2164-8-122)
Supplement: Additional file 8 — Supplementary Figure 3. BP0880 PCR/LDR. [file 1471-2164-8-122-S8.pdf]

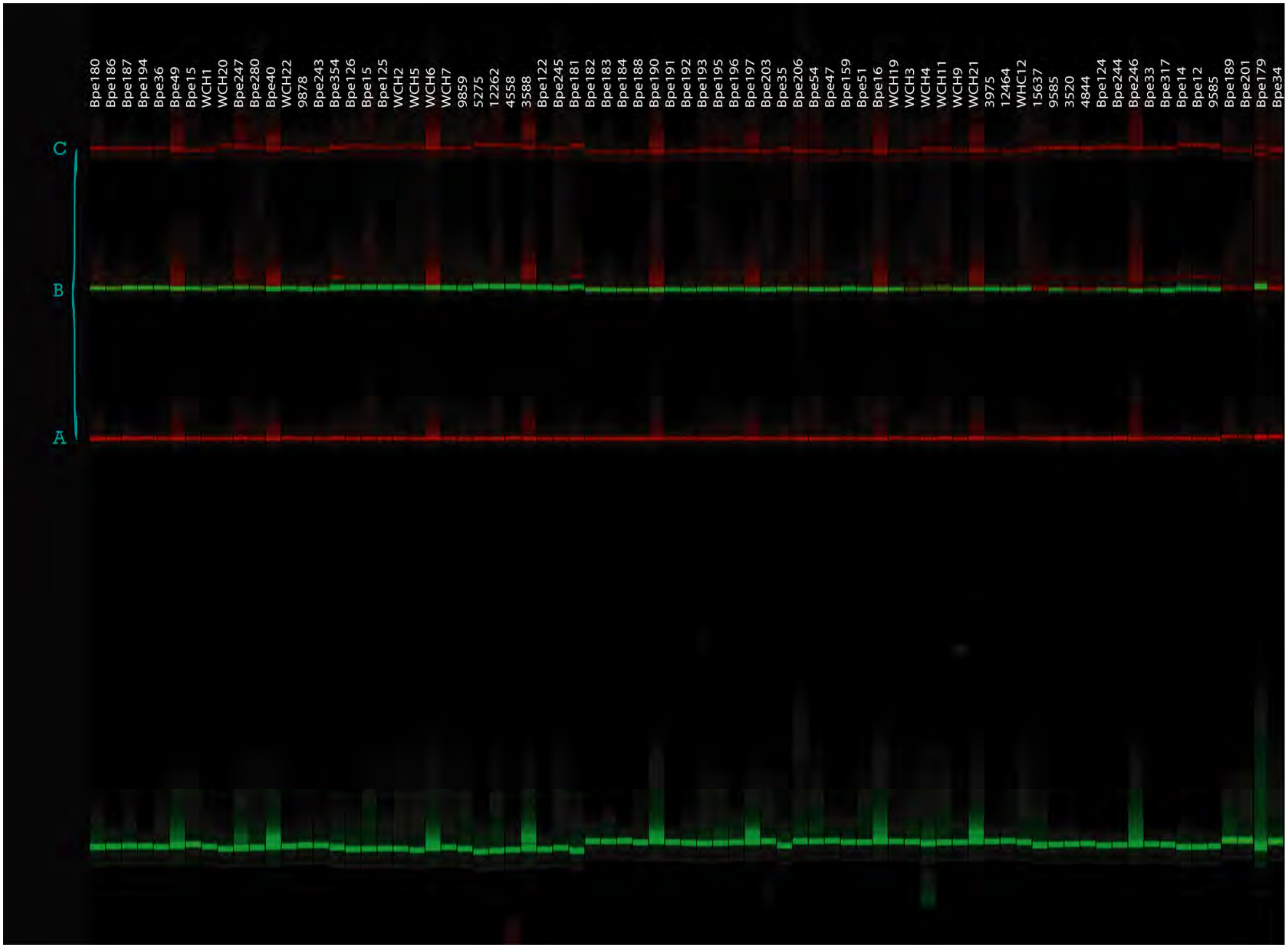

Supplementary Figure 3. BP0880 PCR/LDR
Raw capillary electrophoresis data for ligation products from BP0880 uniplex PCR/LDR (green) and molecular weight standards (red) displayed as if an electrophoretic gel image, with higher molecular weight oligonucleotides closer to the top of the image. Unligated common oligonucleotide is near the bottom of the image, and ligation products are in the size range indicated by the bracket on the left. Each lane represents a single B. pertussis strain (indicated across top). Letters in teal indicate molecular weight standards: A, marker-51; B, marker-67; C, marker-80.
