# Supplementary material for: Phase variation and microevolution at homopolymeric tracts in Bordetella pertussis
Source: BMC Genomics. 2007 May 17;8:122. doi: 10.1186/1471-2164-8-122 (PMC1891110; doi:10.1186/1471-2164-8-122)
Supplement: Additional file 19 — Supplementary Figure 12. Bpe60 colonies screened for bapC HPT length [file 1471-2164-8-122-S19.pdf]

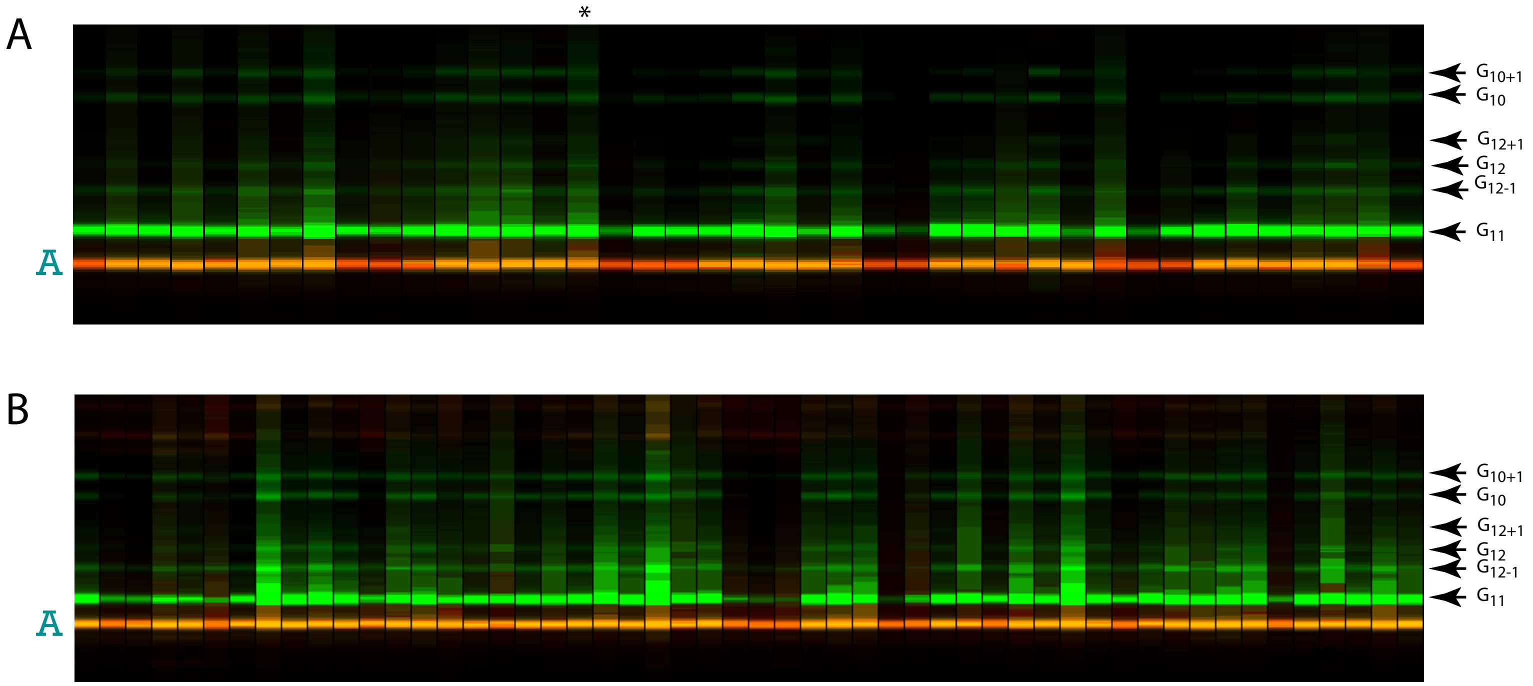

Supplementary Figure 12. Bpe 60 colonies screened for *bapC* HPT length
Raw capillary electrophoresis data for ligation products from *bapC* multiplex PCR/LDR (green) and molecular weight standards (red) displayed as if an electrophoretic gel image, with higher molecular weight oligonucleotides closer to the top of the image. Each lane represents a single pinprick colony of Bpe60. Letters in teal indicate molecular weight standards: A, marker-51; B, marker-67; C, marker-80. (A) 41 colonies from passage one of a single Bpe60 colony. The asterisk denotes the colony that was restreaked on BG blood agar to generate (B) 52 colonies from passage two of a single Bpe60 colony.
